# Parturition Not Necessarily a Painful Process

**Published:** 1872-12

**Authors:** John Stainback Wilson

**Affiliations:** Atlanta, Georgia


*Parturition not Necessarily a Painful Process:* With some Suggestions as to the Hygienic Treatment of Pregnant Women, as a Means of Mitigating the Pains of Labor. By JOHN STAINBACK WILSON, M.D., Atlanta, Georgia.

The proposition that parturition is not necessarily a painful process, will doubtless strike many with astonishment, when viewed in the light of preconceived opinions, which are apparently confirmed by daily observation. Yet, I propose to demonstrate the position—first, physiologically; second, practically or experimentally; and, finally, I will conclude with a notice of the Biblical or theological objections—founded, as I conceive, on an erroneous interpretation of the "curse" inflicted on woman.

My first or physiological argument is, that the nerves of the uterus are derived almost entirely from the organic or sympathetic system, and, therefore, that there is nothing in the nervous structure of the organ to render it highly sensitive when in a normal condition.

Tarnier and other anatomists tell us that the nerves of the uterus are derived from the great sympathetic—some proceeding from the renal, and others from the hypogastric, plexus, with some fibres from the sacral plexus.

Robert Lee, Rendu and Boulard have positively demonstrated that "the whole body of the uterus receives the nerves of organic life exclusively, whilst the nervous apparatus of the neck alone has communications with the spinal nerves."

Carpenter tells us, in his Physiology, that "it is in virtue of the connections of the sympathetic with the cerebro-spinal system, that the parts which are solely supplied with nerves from the former are capable of transmitting sensory impressions to the sensorium." This is asserted of the parts in a normal physiological condition, as the womb is, or should be, in gestation. But the admission is of course made, that parts in a morbid state manifest acute sensibility, the impressions in these cases "seeming (in the language of the writer) to be propagated further than usual—that is, to the sensorium—by virtue of their greater potency."

The conclusion, then, is inevitable, that pain in a part supplied exclusively by the sympathetic system of nerves is no part of the *physiology* of a part so supplied, but is a morbid phenomenon, requiring a strong or long continued impression for its manifestation. The body of the womb, then, being exclusively supplied with nerves of organic life, pain in the body of the womb, when healthy, can form no part in the normal physiological contractions of an uncomplicated labor. Labor is a physiological process; pain does not and cannot belong to physiology, but to pathology. It is a misnomer—a perversion of language—to speak of the physiological pains of parturition. Some attempt to evade this incontrovertible position by the adoption of a fanciful "pathological physiology," as Tarnier, the annotator of Cazeaux, calls it. This writer considers the pains of labor as identical with cramps in the muscles of animal life, and spasmodic contractions of the bowels, bladder, etc. But colic pains are certainly not physiological, but pathological; and if the process of parturition is a strictly physiological one, as all admit, pain, and especially pain in a part endowed with so little sensibility as the body of the womb, must be morbid, and not a normal physiological manifestation. And pray, where else, in the broad domain of physiology, do we find the strange admixture, the painful *physiological* action, which is invoked in explanation of the pains of parturition?

It can hardly be denied that the teachings of all physiological manifestations in other parts, and the nervous structure of the body of the uterus, supplied as it is by the organic system of nerves exclusively, forbid the idea that this part is the seat of pain in the normal contractions of a healthy womb.

But it may be objected, that the argument does not meet the difficulty as to the neck of the os, these parts being supplied, in part at least, by spinal nerves. But admitting that the small supply of the spinal nerves may endow the os and neck with some degree of nervous sensibility, thus rendering the parts so supplied more susceptible to pain and reflex excitement than the body, yet our daily observation teaches us that the neck and os have but very little sensibility in the healthy unimpregnated womb, and that this is true, to a great extent, of those parts in pregnancy. This position is confirmed by Tarnier and numerous other writers; but it is needless to aduce other authorities, when we know that the womb, in a healthy condition, may be handled roughly, and even cauterized, with little or no manifestation of sensibility; and this in many morbid states.

If, then, there is nothing in the nervous structure of the womb to account for the pains of parturition, *how* are these pains produced?

Some writers suppose the pains result from tension of the fibres of the neck; others from pressure on the nerves distributed to the internal surface of the womb; others, again, suppose that they arise from compression of the parts contained within the pelvis, and especially the nervous plexuses; while one writer, already quoted, makes the pains of labor identical with the spasmodic pains of colic; and finally, M. Beau says, that the pains are not in the uterus, but in the lumbo-abdominal nerves.

It will be noticed that *pressure* in some form is regarded as the source of pain in all the theories adduced, unless Tarnier's spasmodic theory be an exception. According to the explanations given, labor is regarded as a mere mechanical act—as the forcible passage of a body through a narrow passage, without considering the *vital* condition and adaptation of the parts through which the passage is to be made.

While pressure doubtless has much to do with the pains of labor, it cannot be the sole, or even, in itself, the principal cause of those pains. It is well known to all obstetricians, that, as Cazeaux says, the pains of *dilatation* are excessive is some women, before the presenting part of the child presses on the uterine orifice. In these cases, we must assume, with the writer quoted, that the pains result from pressure on the almost insensible nerves of the body of the womb; or, I think, we should adopt the much more reasonable conclusion, that a degree of pressure which, in a healthy condition, would be scarcely felt, is greatly aggravated in its effects by a *morbid irritability* of the nervous system in general, and of the sensitive nerves of the os uteri in particular—the difficulty in the os being, in the cases referred to, and in most others, not the *pressure per se*, so much as in the reluctant yielding and stretching of parts deficient in that vital expansibility which certainly belongs to the healthy os, perinæum, and all the soft parts concerned in parturition, and which is certainly necessary for the easy performance of the act. This *vital expansibility* has much, very much, to do with freedom from the pains of labor. And yet, while all writers admit the wonderful adaption of the fœtal parts to the pelvis through which they have to pass, and the wise provisions of nature for the accomplishment of the great work of parturition, still these same writers speak of the act as a mere mechanical one—as the forcible dilatation of ill-constructed parts by a body not adapted to those parts, either in size or shape. Hence, such writers as Desormeaux, Cazeaux, and many others, attribute the dilatation of the os uteri entirely to the wedge-like pressure of the fœtal head, or to the forcible and overpowering contractions of the body of the womb acting on the reluctant fibres of the os.

How much more reasonable, how much more consonant with true physiology, is the explanation of the great physiologist, Carpenter. He says: "It is, in fact, in the contraction of the fibres of the fundus and body of the uterus, and in a relaxation of those about the cervix (which relaxation is something quite different from a mere yielding to pressure, and is obviously a *vital* phenomenon that marks a peculiarity in the action of this part), that the first stage of an ordinary labor essentially consists."

Here, then, we have the whole secret of painful parturition disclosed. The dilatation of the os and associated parts is a vital, and not a mechanical, process; the vital properties of the tissues involved are changed by a morbid or non-physiological condition of those parts, and, as a consequence, secretion is checked or arrested, the tissues are rigid and unyielding, the nervous sensibility is greatly exalted, the organic expansibility is held in abeyance; and the result of all this is, that parturition is translated in truth, as well as in theory, from the domain of physiology to that of pathology and mechanics, and violent overpowering force, attended with atrocious and unnatural pains, is required to overcome the resistance offered by parts morbidly irritable, and wholly or partially deficient in vital expansibility. Hence, the cause of the excessive pains of labor may be summed up in a few words, viz.: morbid irritability of the nervous system in general, and of the uterine nerves in particular, with deficiency or absence of vital expansibility, either as a cause or effect of this nervous irritability. The *degree* of pain will of course be proportioned to the nervous susceptibility; and let it be borne in mind that Cazeaux, M. Beau, and many other writers, admit, after all their explanations, that the degree of pain depends more on this *nervous susceptibility* than on the force of the uterine contractions.

Without stopping to specify the various causes of the nervous irritability which so greatly abound in the present mode of living, and which will readily suggest themselves to every physician, I pass on to my second division, embracing the results of my own observations as to the efficacy of means to prevent or greatly mitigate the pains of labor. Several cases will be very briefly reported, and I ask special attention to the *points* embraced in them, as I cannot stop to enlarge on these.

Case I. Mrs. ——, aged nineteen. First labor eighteen hours; expulsive stage about six hours; pains numerous and severe; no preparatory course of treatment; general health good; previous manner of living tolerably good; development good—no malformation from tight lacing, etc. Second labor twenty-four hours; breech presentation; expulsive stage five hours; no preparatory treatment. Third labor fourteen hours; expulsive stage three hours.

All these labors were pretty severe, but were uncomplicated. After the last she had an attack of phlegmasia dolens, followed by ulceration of the womb, with great debility and impairment of the general health, manifested in palpitation of the heart, dyspepsia, and many distressing nervous symptoms. During this pregnancy, and in previous ones, she suffered from most of the multiform "diseases of pregnancy."

In her fourth pregnancy, she was subjected to the hygienic course yet to be mentioned, and escaped most of the ailments of pregnancy, while parturition was effected in three hours from the beginning, with an almost painless expulsive stage of half an hour, the work being accomplished with only three or four contractions.

In her fifth pregnancy, she had the same or even greater immunity from the diseases of pregnancy than in the fourth, under a treatment similar to the one mentioned. In this she was delivered in three hours from the beginning of labor, with not more than two expulsive efforts, and these were attended with scarcely any pain.

In her sixth labor, dilatation was accomplished in twelve hours, by *painless contractions*, and expulsion was effected in twenty minutes, with six strong and almost painless contractions. And this after a non-child-bearing interval of twelve years, during most of which time she had suffered more or less from ulceration of the womb.

Case 2. Mrs. C., aged about seventeen years; primapara; short stature, firm tissues; closely built, but well formed. She was subjected to a preparatory hygienic course, and was delivered in about six hours from the beginning of labor, with a very short and almost painless expulsive stage. So little complaint was made that I hardly knew when the child was born, though in the room all the time; and the patient did not suppress her complaints, for she expressed surprise that her labor was over so soon, and with so little pain. There was no hemorrhage, scarcely a stain—no bad smell nor other disagreeable accompaniment of child-bed.

Case 3. Mrs. S., aged about eighteen; well formed; good general health. First labor; treatment hygienic during pregnancy; labor six hours; expulsive stage short—only one or two contractions finished it, and she expressed herself as feeling scarcely any pain. Moved in two weeks, on cars.

In her second pregnancy, she followed the hygienic treatment, and was delivered in three or four hours from the beginning of labor; expulsive stage about twenty minutes, and almost painless. No accident; recovery good.

In her third labor, she was delivered in three-quarters of an hour from the first pain. In this case, there were but nine contractions in all; seven effected dilatation, and two, which were scarcely felt, accomplished expulsion.

In her fourth pregnancy, she was traveling in a wagon, and could not use hygienic means. In this, her labor was twelve hours long, but other particulars are not known.

Case 4. Mrs. F., aged twenty-five or thirty; good health; well formed; third labor. Reports other labors severe. Treatment in this (the third) hygienic. Labor four or five hours; expulsive stage about half an hour; little pain; no accidents of any kind.

Case 5. Mrs. H., primapara, aged about eighteen; form and general health good, but she had been delicately raised. Treatment as in other cases. Labor six hours; expulsive stage three-quarters of an hour; scarcely any pain; no hemorrhage, or other disagreeable symptom.

These cases need no comment; I will only call attention to some of the prominent points.

The first case embraces six labors—three with hygienic treatment, and three without. The latter were respectively eighteen, twenty-four and fourteen hours in duration, and by no means without pain. The last three labors of the same woman, under hygienic treatment, were almost entirely painless; and two of these were accomplished in three hours, while the other, though occupying twelve hours in dilatation, was unattended by pain in this stage; while expulsion was effected in the marvelously short space of twenty minutes. And this, be it remembered, after a sterile interval of twelve years, as a consequence of grave uterine disease.

The second and fifth cases were primaparæ. These cases will meet the objection that, however it may be with others, *young* women *must* suffer very considerably in a first labor.

The third case is also one of a first labor; and this was only six hours in duration, and almost painless; while of two others treated hygienically, the labor was three or four hours long in one, and the other only three-quarters of an hour, and in each case almost entirely painless.

The short period for the accomplishment of parturition in some of these cases proves that a long time is not a necessary factor in a safe delivery, but that when the parts are fully prepared—when the vital expansion and the expulsive contractions proceed *pari passu*—the great act of parturition can be effected even in twenty or thirty minutes, with perfect freedom from accidents, and with but little pain.

If further evidence is needed in proof of the position that parturition is not necessarily a painful process, we have it in the numerous examples of absolutely painless labors to be found in the reports of almost every obstetric writer. And again, we have conclusive evidence on this point, and also demonstration of the effects of modes of life on parturition, by comparing the process among women in the different classes of society.

While all civilized child-bearing women cannot be said to be in a state of actual open disease, there cannot be a shadow of doubt that their habits in civilized life tend greatly to beget that morbid irritability and that preternatural susceptibility to pain which have so much to do with the pangs of parturition. We all know the difference between the parturitions of women in civilized and savage life. We are all familiar with the fact that Indian women really have no lying-in; that they "fall behind for a little on their journeys through the forest, deliver themselves, and shortly make up to their husbands, and continue their journey with their offspring on their back." Among the South American Indians, "a mother, immediately on her delivery, takes her child, and going down to the nearest stream of water, washes herself and it, and returns to the usual labors of her station." And even in civilized life, we all know that the pains and dangers of child-birth are diminished in proportion as women approach plain, healthful, natural habits of living. Those who are in moderate circumstances, who are equally removed from the depressing influences of extreme poverty and the enervating, destructive indulgences purchased by wealth, have easier labors and more speedy recoveries than either of the extremes of society; and the poorest often escape the "primal curse" much better than the pampered daughter of idleness and luxury. Hence, the negro women of the Southern States, who were compelled to lead an active life, and to live on plain food, with minds undisturbed by corroding cares, had, as every Southern physician knows, much easier labors, and were much less exposed to so-called accidents, than their mistresses.

Now, nature being governed by fixed and uniform laws in all her operations, if it can be proved that even one woman, in a natural condition, without the aid of any anæsthetic, has been delivered with little or no pain, the conclusion is inevitable, that parturition is *not* necessarily a painful process. It is admitted that not only one, but many, have been thus delivered. Why, then, may not all healthy, well-formed women have comparatively, or even positively, painless labors by obedience to the laws of health? There is nothing in the anatomical structure of woman to forbid such an idea. On the contrary, I have shown that the nervous structure of the womb is of such a nature as to render this organ peculiarly insusceptible to pain; and while I have not stopped to elaborate the point, all are ready to admit the wise provisions of nature for the expulsion of the fœtus by means of the beautiful mechanical arrangement of the pelvic bones.

But while anatomy, physiology, and numerous facts, sustain the idea that great pain is not a necessary part of normal parturition, the Bible is appealed to as conclusive evidence to the contrary, and the "primal curse" is invoked as an unanswerable argument. I believe in the Bible as a rule of action, as an authority in morals, and as a system of spiritual truths and doctrines; but I do not believe in that mistaken piety which would array the Bible in opposition to every discovery in science. I do not think that this book was ever intended to teach anatomy, physiology, astronomy, geology, or any of the sciences. Yet, it is part of my creed, that there is nothing in the Bible incompatible with the teachings of true science, when the book is confined to its legitimate sphere, and properly understood and interpreted. What, then, of that *curse*—that *dreadful* CURSE—"In sorrow shalt thou bring forth children"? This is often quoted, "*in pain*," etc. But the word sorrow does not necessarily include physical pain, and we might find abundant sources of sorrow for Eve in her mental condition, in the anxieties and gloomy forebodings that would naturally fill her mind on the birth of a child into a world alienated from God and cursed with the terrible evils of sin. But, not to take advantage of a verbal quibble, I freely admit that many portions of the Bible express, in strong terms, physical suffering in connection with parturition. A few of these may be given: "Fear took hold of them there, and pains, as of a woman in travail." "They shall be in pain, as a woman that travaileth." "Therefore are my loins filled with pain; pangs have taken hold of me, as the pangs of a woman that travaileth." These quotations might be greatly multiplied, for the Bible has many such expressions.

It is admitted, then, that women suffer, grievously suffer, in parturition, and have thus suffered since the introduction of sin into the world; but I contend that these sufferings are not due to any original defect in the physical organization of woman, nor to the direct effect of the curse inflicted on her, but to the violation of physiological laws, as a consequence of the introduction of sin into the world. The physical organization of woman must have been perfect, as she emanated from the plastic hand of the Almighty.

And again, no one can doubt that the first woman had the same system of nerves, muscles and bones as the women of the present day; and therefore, if the first woman could have been delivered without pain, in the perfection of her physical organization, before the introduction of sin, so could she and her daughters continue to bear children without pain, unless their physical organization had been deteriorated by violations of the laws of their being.

Surely no one will be so absurd as to assume that God made any change in the anatomy or physiology of man or woman by pronouncing a curse on either. The natural or physiological laws, then, were unchangeable from the beginning—were instituted before the fall, and were not changed in a single "jot or tittle" by the fall, but remain in all their integrity and force to the present time. But while the laws which govern man's physical being are not changed, his *relation* to those laws *is* changed by reason of sin, which latter causes him to contravene those laws in numberless ways. And thus does he suffer the penalty which follows as an unavoidable sequence of his violations of physiological laws; and he or she, the man or the woman, suffers individually, reasonably, legitimately, and righteously, in this way and not as a *whole race*, through the mysterious and inexplicable operation of a harsh, indiscriminate and vindictive curse. Whenever men and women cease to transgress the laws of their organism—whenever they, by obedience to those laws, place themselves in proper relation to them—then they are visited with blessings, and not with curses; then the terrible curse on woman is no longer operative, and she can fulfill the great command to "multiply and replenish the earth," without pain, as the perfection of her unimpaired and unperverted physical organization abundantly qualifies her to do.

The curse on woman, then, must be regarded as a simple declaration—as a prediction of the evils that would befall her by reason of the violations of physiological laws, as a consequence of sin. All the evils resulting from such violations were foreseen by Divine Wisdom. They were announced beforehand, and they have been literally fulfilled, not by the *direct* withering influence of a curse, but by the voluntary, self-inflicted agency of woman, who still remains under the great primal law of the race—*obey and live, disobey and die.*

This law of *works* still prevails in the physical and physiological world. For moral transgressions we have an atonement, but there is no atonement for violations of physiological laws. The penalty *must* follow the infraction. Were it otherwise, the whole wise economy of the universe would be subverted, and still greater evils than those that now exist would befall the human race, by the removal of all salutary restraints.

How much more reasonable such doctrines as these—how much more accordant with Divine Wisdom—than the teachings of a misguided theology, which would make all the evils that afflict the human family the direct consequences of a curse inflicted on our first parents—a curse whose consequences can by no possibility be avoided!

Having, as I trust, established my first position—that parturition is not necessarily a painful process—I next proceed to notice, briefly, the means by which the pains of labor may be mitigated. This I must do in rather general terms, as it would prolong this paper too much to enter into the details of all the points involved.

To fulfill the great and all-important indication of removing that morbid irritability, and that excessive rigidity which are the great causes of the pains of labor, we should avail ourselves of every agent which will place the system in a natural, healthy condition; and as this morbid irritability is doubtless superinduced by bad habits of living in the majority of cases, the first step is the correction of those habits by the adoption of a natural, healthy mode of living. This, in most cases, will involve an entire change in the whole manner of life.

The pregnant woman, instead of taking an extra quantity of food, because "she has to eat for two," should eat only so much as the stomach can easily manage. Her diet should be mostly of the vegetable class, and cooling and laxative in its nature, avoiding all highly-seasoned and stimulating articles. The same rule applies to drinks, even to the exclusion of those common beverages, tea and coffee.

It is hardly necessary to urge on the attention of a body of physicians the importance of an abundance of pure air and sunlight—of properly-regulated exercise—of sufficient sleep—of perfectly loose clothing, and of mental and emotional quietude, during the whole period of gestation. On these points I will only say that pure air, so necessary to health under all circumstances, is especially great during the latter stages of pregnancy, when the diaphragm is pushed upward by the ascending womb, thus diminishing the capacity of the lungs, while the liver and great blood-vessels are compressed, the whole circulation is oppressed, and the secretions and excretions are checked or suppressed.

Of exercise it is sufficient to say, that it is only another name for life itself; for life is motion—constant, ceaseless motion. Exercise promotes all the vital movements, and stands in direct antagonism to stagnation, disease and death.

As to the advantages and necessity of light, we can form some conception of its importance to animal life by considering its power in reversing the chemical and vital changes of vegetable life. It is well known that through the agency of light, vegetation absorbs carbon and gives off oxygen in the day, while at night just the reverse is true. The influence of the absence of light over vegetation is again manifest in rendering plants excluded from it, colorless, sickly, and brittle.

Of sleep, I will only say that the excitability of the nervous system of pregnant women, and the extraordinary drafts made on them, urgently demand an extra amount of sleep during gestation.

On clothing, it is needless to enlarge. The terrible evils arising from tightly-fitting dresses being admitted under all circumstances, the *murderous* effects of all kinds of constriction and compression in pregnancy are obvious.

It is equally unnecessary to dwell on the importance of the mental hygiene of pregnant women; suffice it to say that of all human beings, women, and especially child-bearing women, are under the greatest, the most sacred obligation to obey all the laws which govern their mental and moral constitutions.

Having thus hastily passed in review the principal hygienic agents to which we should give attention in pregnancy, there is one other agent which merits a more detailed notice, because I have reason to believe that its importance is not duly estimated by physicians. I allude to bathing. On this point I cannot do better than to extract from a published essay, read before the Atlanta Academy of Medicine.*

"To subdue the nervous and vascular excitement incident to pregnancy, and to depurate the blood, there is nothing so safe and effectual as tepid, cool, or even cold baths. These baths abstract excessive heat, equalize the circulation, remove internal congestions, and strengthen the whole system—thus preparing it to furnish suit-

* This essay treats more fully on the points above alluded to, and on others pertaining to the hygiene of gestation, than can be done in this paper.—*Vide Atlanta Medical and Surgical Journal*, December, 1871.

able elements for the new being, and to pass safely through the critical time of parturition. The feelings of the patient may be safely consulted as to the temperature of the water. The sponge-bath, or wash-down, is the best mode of applying it. Of course, the shower bath, and all applications that cause a violent shock, should be avoided. Pregnant women generally bear cold water remarkably well, but they cannot well tolerate violent impressions of any kind. The sponge bath should be used every day, or every other day, from the beginning of pregnancy, and the hip-bath should be resorted to once a day during the last month or two. The temperature of this should be moderate, and it should not be continued more than five or ten minutes each time. The cold hip-bath, with the general baths recommended, places the system in the most favorable condition for parturition, and, I may add, is one of the most, if not the most, effectual of all the means at our command for preventing or allaying that morbid irritability and that excessive rigidity which are the great, the principal, if not the sole, causes of the pains of labor."

While, in the cases of remarkably easy parturition reported in this essay, other means were not neglected, I must say that the gratifying results in these cases were, in my view, due more to judicious bathing than to any other or all other agencies. Yet, everything should be brought into requisition that will equalize, the circulation, promote the secretions and excretions, relieve oppression, hasten the vital, eliminative and nutritive functions, and allay nervous irritability. The principal means may be thus summed up: Good, digestible, laxative diet, in moderate quantities; cooling, bland, unstimulating drinks; avoidance of stooping or doubled positions; loose, comfortable clothing; pure air by day and night; abundant sleep; equanimity of mind; sunlight; and last, though not least, general and local baths, followed by friction of the skin.

Instead of multiplying details of treatment, I think I cannot do better than to conclude with the great principle which, I think, should govern and guide us in the management of pregnancy. It is this: *Pregnancy, though a natural physiological condition, is one of increased excitement, attended with extraordinary demands on the whole organism.* Bearing this principle in mind, and ever remembering that the great cause of the pains of parturition is a morbid irritability of the nervous system, attended with excessive rigidity or deficiency of vital expansibility, the proper treatment will readily suggest itself to every intelligent physician.—*Transactions of Georgia Medical Association.*

